# Metabolically ‘extremely unhealthy’ obese and non-obese patients with diabetes and the risk of cardiovascular events: a French nationwide cohort study

**DOI:** 10.1007/s00392-023-02344-8

**Published:** 2023-12-04

**Authors:** Katarzyna Nabrdalik, Arnaud Bisson, Krzysztof Irlik, Gregoire Fauchier, Pierre Henri Ducluzeau, Gregory Y. H. Lip, Laurent Fauchier

**Affiliations:** 1 Liverpool Centre for Cardiovascular Science at University of Liverpool, Liverpool John Moores University and Liverpool Heart and Chest Hospital, Liverpool, UK; 2 Faculty of Medical Sciences in Zabrze, Department of Internal Medicine, Diabetology and Nephrology, Medical University of Silesia, Katowice, Poland; 3 Service de Cardiologie, Centre Hospitalier Universitaire et Faculté de Médecine, Université de Tours, Tours, France; 4 Service de Cardiologie, Centre Hospitalier Universitaire d’Orléans, Orléans, France; 5 Faculty of Medical Sciences in Zabrze, Student’s Scientific Association at the Department of Internal Medicine, Diabetology and Nephrology, Medical University of Silesia, Katowice, Poland; 6 Service de Médecine Interne, Unité dʼEndocrinologie Diabétologie et Nutrition, Centre Hospitalier Universitaire et Faculté de Médecine, Université de Tours, Tours, France; 7 INRA, UMR 85, Unit SENSOR, Nouzilly, France; 8 Danish Center for Health Services Research, Department of Clinical Medicine, Aalborg University, Aalborg, Denmark

**Keywords:** Cardiovascular events, Diabetes, Extremely unhealthy, Non-obese

## Abstract

**Background:**

Non-obese patients with diabetes mellitus (DM) are becoming more prevalent, but their cardiovascular risk (CV) especially when accompanied with cardio-renal-metabolic co-morbidities (hypertension, chronic kidney disease, hyperlipidemia) is not well characterised. The aim of the study was to assess the CV risk among patients with DM in relation to obesity and cardio-renal-metabolic co-morbidities.

**Materials and methods:**

This was a cohort study of all patients with DM without a history of major adverse cardiovascular event who were hospitalized for any reason in France in 2013 with at least 5 years of follow-up. They were categorized by the presence of obesity vs no obesity, as well as three cardio–renal–metabolic co-morbidities: hypertension, chronic kidney disease, hyperlipidemia. ‘Extremely unhealthy’ patients with DM were defined as those having all 3 co-morbidities.

**Results:**

There were 196,112 patients (mean age 65.7 (SD 13.7) years; 54.3% males) included into the analysis. During a mean follow-up of 4.69 ± 1.79 years, when adjusted for multiple covariates, the non-obese and ‘extremely unhealthy’ obese patients had the highest risk of CV death [aHR 1.40 (95% CI, 1.22–1.61) and 1.48 (95% CI, 1.25–1.75), respectively]. The ‘extremely unhealthy’ obese had the highest risk of MACE-HF [aHR 1.84 (95% CI, 1.72–1.97)] and new-onset AF [aHR 1.64 (95% CI, 1.47–1.83)].

**Conclusion:**

Both non-obese and obese patients with DM with associated cardio-renal-metabolic co-morbidities are an ‘extremely unhealthy’ phenotype with the highest risk of CV death and CV events.

**Graphical abstract:**

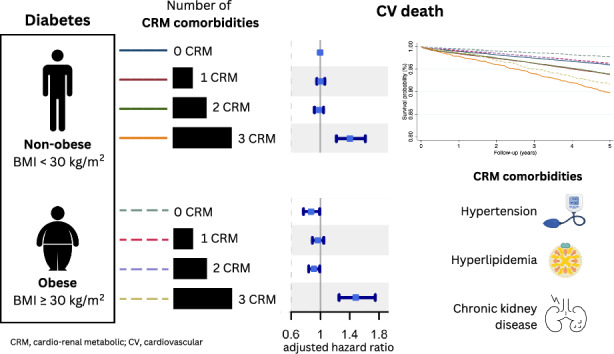

**Supplementary Information:**

The online version contains supplementary material available at 10.1007/s00392-023-02344-8.

## Introduction

During the last decades, cardiovascular disease (CVD) risk is increased in obese patients with diabetes mellitus (DM) [1]. However, nowadays, there is an increasing prevalence of type 2 DM (T2DM) with lean/normal body mass index (BMI), which was for decades thought to be the minority of patients with DM [2]. In a recent study from the United States, ‘lean’ populations showed an approximately ninefold higher growth in the prevalence of DM over the last 5 years, when compared with overweight/obese populations [3]. Indeed, the metabolically obese normal weight (MONW) phenotype was first described more than 40 years ago [4], yet T2DM with normal weight remains an understudied DM phenotype.

There is the so-called ‘obesity paradox’ indicating that there is reduced mortality or incidence of CV events in patients with obesity comparing to the ones who are not obese [5–8]. The obesity paradox may be present in patients with DM [9–13], but not all the studies have reported the same outcomes [14, 15]. However, the impact of obesity in DM may not be binary (i.e. yes/no), given the comorbidities associated with DM. Indeed, the contradictory results in relation to the obesity paradox in patients with DM may be linked to different populations studied and those with and without coronary artery disease. In addition, the CV risk in obesity vs no obesity may be intimately linked to cardio-renal-metabolic co-morbidities i.e. hypertension, chronic kidney disease (CKD) and hyperlipidemia.

In this longitudinal nationwide French cohort study, we aimed to evaluate CV risks in patients with DM in relation to the presence of obesity vs no obesity, as well as the presence of one or more concomitant cardio-renal-metabolic co-morbidities.

## Research design and methods

### Study design

This was a longitudinal cohort study based on the database of national hospitalization which covered hospital care for the entire French population. The details of this study population was described elsewhere [16]. In brief, the data was collected from the national administrative Programme de Médicalisation des Systèmes d’Information (PMSI) for all patients admitted to French hospitals for any reason in the year 2013 with at least 5 years of complete follow-up (unless death). The following data has been collected: demographics, diabetes mellitus (DM), obesity and any other co-morbidities, medical history and events during hospitalization and follow up. Each diagnosis was identified with International Classification of Diseases tenth revision (ICD-10) codes. Diabetes was identified with the following ICD-10 codes: E10 for diabetes type 1 (T1DM), E11 for diabetes type 2 (T2DM), E13 and E14 for other types of DM, and obesity was identified with ICD-10 code E65. Exclusion criteria were as follows: age < 18 years, previous hospitalization for myocardial infarction (MI), ischemic stroke (IS) or new-onset HF (major adverse cardiac event [MACE]-HF) as well as atrial fibrillation (AF) recorded during 2010–2013, or underweight and malnutrition (identified with the following ICD-10 codes: E41, E43, E44, E46, F50.8, K91.2 and R63.6), and those without DM.

### Cardio-reno-metabolic phenotypes

We divided the cohort into eight groups depending on the presence of obese/non-obese phenotype, and the number of cardio-renal-metabolic comorbidities defined as hypertension (HA) and/or CKD and/or hyperlipidemia with their respective ICD-10 codes (Supplementary Table 1). CKD was incorporated as a cardio-renal-metabolic comorbidity alongside hypertension and hyperlipidemia, to offer a comprehensive understanding of CV risks. This inclusion is predicated on CKD being considered a modifiable risk factor, as its progression and associated CV risks can be mitigated through targeted pharmacological interventions. Group 1, group 2 and group 3 consisted of non-obese patients with DM and zero, one or two other cardio-reno-metabolic comorbidities (ie. hypertension, CKD, hyperlipidemia) respectively; Group 4 was categorised as “non-obese extremely unhealthy” consisting of non-obese patients with DM and 3 additional cardio–renal–metabolic co-morbidities. Group 5, group 6 and group 7 consisted of patients with obesity, DM and 0, 1 or 2 other cardio-renal-metabolic co-morbidities respectively. Group 8 were “extremely unhealthy obese” consisting of obese patients with DM plus 3 additional cardio-reno-metabolic comorbidities.

### Outcomes

The primary outcome was the occurrence of all-cause death, MACE-HF (CV death, MI, IS, new-onset HF) and new-onset AF during follow up. We identified these outcomes with their respective ICD-10 codes. The patients were followed from the first hospitalization in 2013 till 31 December 2019 or until death.

Information on outcomes during the follow-up was obtained by analyzing the PMSI codes for each patient. The mode of death (CV or non-CV) was identified based on the main diagnosis during hospitalization resulting in in-hospital death. Patients were also analyzed according to number of cardio–renal–metabolic comorbidities namely patients with zero additional abnormalities were those with DM (obese or non-obese) excluding hypertension, CKD and hyperlipidemia after which, we investigated DM patients with 1, 2 or 3 additional comorbidities.

### Ethics

The medical information used in the database was anonymized, and the analysis was conducted retrospectively. Therefore, neither patient consent nor ethics committee approval was required for this study. The study was however approved by the institutional review board of the Pole Coeur Thorax Vaisseaux from the Trousseau University Hospital (Tours, France) on 1 December 2015, and registered as a clinical audit. The French Data Protection Authority granted access to the PMSI data. Moreover, procedures for data collection and management were approved by the Commission Nationale de lʼInformatique et des Libertés (CNIL), the independent National Ethical Committee protecting human rights in France, which ensures that all information is kept confidential and anonymous, in compliance with the Declaration of Helsinki (authorization number 1897139).

### Statistical analysis

Qualitative variables are presented as frequency and percentages and quantitative variables as means (standard deviations [SDs]). Comparisons were made using chi-square tests for categorical variables and Studentʼs t test for continuous variables. Incidence rates (IRs) with 95% confidence interval (95% CI) were calculated for outcomes of interest in each of four subgroups. A multivariable analysis for clinical outcomes during the whole follow-up in each subgroup was performed using a Cox regression model to calculate the adjusted hazard ratio (aHR) and 95% CI for each subgroup, the reference category were individuals with DM and normal weight (zero additional cardiorenal–metabolic comorbidities). In the first model we adjusted for age at baseline and sex, while in the second model we adjusted for the following variables: age, sex, type of diabetes, smoking status, alcohol-related diagnoses, valve disease, coronary artery disease, previous percutaneous coronary intervention, previous coronary artery bypass grafting, vascular disease, previous pacemaker or implantable cardioverter defibrillator, lung disease, sleep apnoea syndrome, liver disease, thyroid disease, inflammatory disease, anaemia, previous cancer, cognitive impairment and illicit drug use. Kaplan–Meier curves were plotted with the duration from the enrollment to either the last follow-up date or occurrence of outcome of interest. All analyses were performed with STATA version 16.1 (Stata Corp., College Station, TX, USA).

## Results

**Fig. 1:**
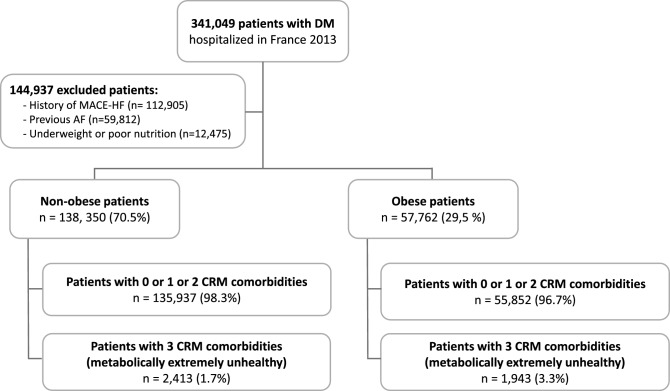
Flowchart of the study. *AF* atrial fibrillation, *CRM* cardio-renal-metabolic, *DM* diabetes mellitus, *MACE-HF* composite of CV death, ischemic stroke; myocardial infarction and new-onset heart failure

Of 3,381,472 patients hospitalized in France in the year 2013 we included 341,049 patients who had DM in this analysis. Subsequently, we excluded those with previous MACE-HF (112,905 patients), previous AF (59,812 patients) and underweight or under nutrient (12,475 patients). We finally included 196,112 patients (mean age 65.7 (13.7) years; 54.3% male) (Fig. 1).

Among these individuals, 138,350 (70.5%) were classified as non-obese and 57,762 (29.5%) as obese subjects. Population characteristics at baseline showed significant differences across all compared features between eight subgroups of individuals with obesity or no obesity, based on the number of cardio–renal–metabolic comorbidities (Table 1). Those with more CV comorbidities were older, with higher proportion of males and higher rate of non-metabolic comorbidities than those without.

**Table 1:** Baseline characteristics of diabetes mellitus patients according to body size phenotypes and metabolic status

	Overall *n* = 196,112	Non obese patients *n* = 138 350 (70.5%)				Obese patients *n* = 57 762 (29.5%)				*p* Value
		0 CRM comorbidities *n* = 49,832 (36.0%)	1 CRM comorbidities *n* = 55,219 (39.9%)	2 CRM comorbidities *n* = 30,886 (22.3%)	3 CRM comorbidities *n* = 2413 (1.7%)	0 CRM comorbidities *n* = 10,460 (18.1%)	1 CRM comorbidities *n* = 22,822 (39.5%)	2 CRM comorbidities *n* = 22,540 (39.0%)	3 CRM comorbidities *n* = 1940 (3.3%)	
Age (year)	65.7 ± 13.7	61.6 ± 16.5	70.0 ± 12.0	69.6 ± 11.0	68.6 ± 12.3	57.2 ± 14.5	63.8 ± 12.0	64.6 ± 10.4	66.5 ± 10.5	< 0.001
Sex (male)	106,430 (54.3)	28,206 (56.6)	31,215 (56.5)	18,759 (60.7)	1535 (63.6)	4336 (41.5)	10,161 (44.5)	11,205 (49.7)	1013 (52.2)	< 0.001
Type 1 diabetes	25,092 (12.8)	11,810 (23.7)	5953 (10.8)	3037 (9.8)	326 (13.5)	1133 (10.8)	1458 (6.4)	1261 (5.6)	114 (5.9)	< 0.001
Type 2 diabetes	168,406 (85.9)	36,988 (74.2)	48,552 (87.9)	27,493 (89.0)	2049 (84.9)	9191 (87.9)	21,157 (92.7)	21,162 (93.9)	1814 (93.5)	< 0.001
Other types of diabetes	2614 (1.3)	1034 (2.1)	714 (1.3)	356 (1.2)	38 (1.6)	136 (1.3)	207 (0.9)	117 (0.5)	12 (0.6)	< 0.001
Hypertension	121,777 (62.1)	0 (0.0)	45,654 (82.7)	30,738 (99.5)	2413 (100)	0 (0.0)	18,569 (81.4)	22,463 (99.7)	1940 (100)	< 0.001
Hyperlipidemia	64,454 (32.9)	0 (0.0)	8328 (15.1)	26,666 (86.3)	2413 (100)	0 (0.0)	4007 (17.6)	21,100 (93.6)	1940 (100)	< 0.001
Chronic kidney disease	11,721 (6.0)	0 (0.0)	1237 (2.2)	4368 (14.1)	2413 (100)	0 (0.0)	246 (1.1)	1517 (6.7)	1940 (100)	< 0.001
Smoker	16,770 (8.6)	3460 (6.9)	3913 (7.1)	3158 (10.2)	285 (11.8)	922 (8.8)	2027 (8.9)	2776 (12.3)	229 (11.8)	< 0.001
Alcohol-related diagnoses	14,527 (7.4)	4211 (8.5)	4151 (7.5)	1952 (6.3)	145 (6.0)	722 (6.9)	1641 (7.2)	1575 (7.0)	130 (6.7)	< 0.001
Valve disease	5275 (2.7)	748 (1.5)	1543 (2.8)	1248 (4.0)	108 (4.5)	157 (1.5)	632 (2.8)	750 (3.3)	89 (4.6)	< 0.001
Coronary artery disease	24,108 (12.3)	3003 (6.0)	6252 (11.3)	6426 (20.8)	596 (24.7)	543 (5.2)	2427 (10.6)	4353 (19.3)	508 (26.2)	< 0.001
Previous PCI	5231 (2.7)	656 (1.3)	1300 (2.4)	1634 (5.3)	111 (4.6)	92 (0.9)	435 (1.9)	915 (4.1)	88 (4.5)	< 0.001
Previous CABG	561 (0.3)	36 (0.1)	110 (0.2)	191 (0.6)	16 (0.7)	8 (0.1)	47 (0.2)	140 (0.6)	13 (0.7)	< 0.001
Vascular disease	24,697 (12.6)	2835 (5.7)	6525 (11.8)	6850 (22.2)	814 (33.7)	520 (5.0)	2202 (9.6)	4343 (19.3)	608 (31.3)	< 0.001
Previous pacemaker or ICD	3743 (1.9)	620 (1.2)	1333 (2.4)	848 (2.7)	75 (3.1)	69 (0.7)	348 (1.5)	396 (1.8)	54 (2.8)	< 0.001
Lung disease	23,113 (11.8)	4029 (8.1)	5931 (10.7)	3311 (10.7)	260 (10.8)	1504 (14.4)	3964 (17.4)	3783 (16.8)	331 (17.1)	< 0.001
Sleep apnoea syndrome	18,166 (9.3)	1276 (2.6)	2438 (4.4)	1646 (5.3)	140 (5.8)	1626 (15.5)	4899 (21.5)	5630 (25.0)	511 (26.3)	< 0.001
Liver disease	15,124 (7.7)	3270 (6.6)	3566 (6.5)	1767 (5.7)	136 (5.6)	1001 (9.6)	2407 (10.5)	2809 (12.5)	168 (8.7)	< 0.001
Thyroid diseases	17,375 (8.9)	2970 (6.0)	4110 (7.4)	2776 (9.0)	245 (10.2)	1037 (9.9)	2674 (11.7)	3275 (14.5)	288 (14.8)	< 0.001
Inflammatory disease	10,720 (5.5)	2154 (4.3)	2942 (5.3)	1809 (5.9)	241 (10.0)	502 (4.8)	1345 (5.9)	1538 (6.8)	189 (9.7)	< 0.001
Anaemia	20,523 (10.5)	3758 (7.5)	5721 (10.4)	4116 (13.3)	874 (36.2)	717 (6.9)	2264 (9.9)	2454 (10.9)	619 (31.9)	< 0.001
Previous cancer	31,200 (15.9)	7403 (14.9)	10,469 (19.0)	5327 (17.2)	376 (15.6)	1174 (11.2)	3322 (14.6)	2858 (12.7)	271 (14.0)	< 0.001
Cognitive impairment	5760 (2.9)	1326 (2.7)	2347 (4.3)	1070 (3.5)	93 (3.9)	110 (1.1)	413 (1.8)	353 (1.6)	48 (2.5)	< 0.001
Illicit drug use	650 (0.3)	278 (0.6)	132 (0.2)	61 (0.2)	9 (0.4)	35 (0.3)	80 (0.4)	51 (0.2)	4 (0.2)	< 0.001

Values are *n* (%) or mean ± SD

*CABG* coronary artery bypass grafting, *CRM* cardio–renal–metabolic, *ICD* Implantable cardioverter defibrillator, *PCI* Percutaneous coronary intervention

### Mortality and CV outcomes

During a mean follow-up of 4.69 ± 1.79 years (median [IQR]: 5.47[4.74–5.82]) 61,787 (31.5%) patients died, including 10,981 (5.6) who died due to CV causes (Supplementary Table S4). The highest incidence of all-cause death and CV death, IS and MI was observed for the group of non-obese patients with three comorbidities (10.2%, 2.5%, 3.4%, 2.8% respectively). Both patients with obesity as well as those without obesity — but with 3 concomitant cardio-renal-metabolic abnormalities presented with the highest incidence of MACE-HF (16.5% and 16.2%) and new-onset AF (5.4% and 5.4%).

**Fig. 2:**
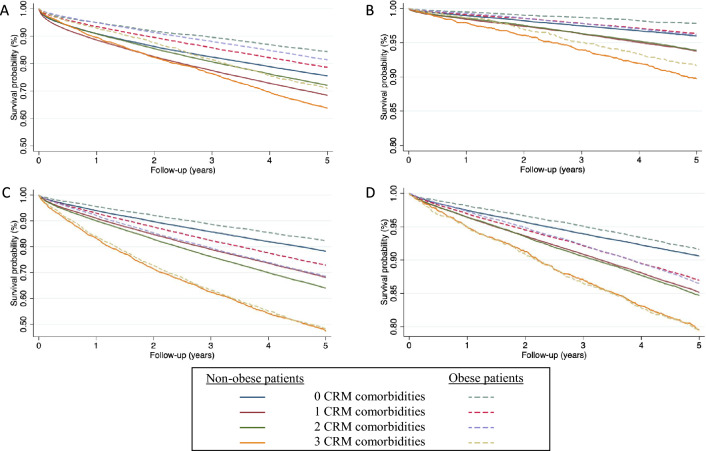
Kaplan–Meier curves of all cause-death (**A**), CV death (**B**), MACE-HF (**C**), and new-onset AF (**D**). Log rank *p* values all < 0.0001. *AF* atrial fibrillation, *CRM* cardio-renal-metabolic, *CV* cardiovascular, *MACE-HF* composite of cardiovascular death, ischemic stroke, myocardial infarction and new-onset heart failure

Obese patients with no additional cardio-renal-metabolic comorbidities besides DM had the lowest incidences of all-cause death, CV death, MACE-HF and AF (3.9%; 0.6%; 4.6% and 2.1%, respectively), even lower than non-obese without metabolic comorbidities (6.2%; 0.9%; 5.8% and 2.4%). Incidence rates per patient-year of major adverse events are presented in Table 2 with corresponding Kaplan–Meier survival curves shown in Fig. 2 (all Log-rank < 0.0001).

**Table 2:** Incidence rates (%/year) of major adverse events according to body size phenotypes and number of metabolic abnormalities

	Non-obese				Obese			
	0 CRM comorbidities	1 CRM comorbidities	2 CRM comorbidities	3 CRM comorbidities	0 CRM comorbidities	1 CRM comorbidities	2 CRM comorbidities	3 CRM comorbidities
% IR (95% CI)								
All-cause death	6.19 (6.09–6.29)	8.49 (8.37–8.60)	7.47 (7.32–7.61)	10.15 (9.56–10.77)	3.86 (3.69–4.02)	5.46 (5.32–5.60)	4.81 (4.68–4.94)	8.04 (7.48–8.65)
CV death	0.94 (0.90–0.98)	1.49 (1.44–1.54)	1.5 (1.44–1.57)	2.49 (2.20–2.80)	0.56 (0.50–0.63)	0.91 (0.86–0.97)	0.94 (0.89–1.00)	2.06 (1.78–2.37)
MACE-HF	5.8 (5.7–5.91)	9.09 (8.96–9.22)	10.48 (10.29–10.66)	16.46 (15.59–17.37)	4.64 (4.46–4.84)	7.48 (7.31–7.65)	8.82 (8.63–9.01)	16.19 (15.25–17.18)
New-onset AF	2.38 (2.31–2.44)	3.89 (3.81–3.97)	4.02 (3.92–4.13)	5.41 (4.97–5.89)	2.13 (2.01–2.26)	3.41 (3.3–3.53)	3.5 (3.39–3.62)	5.43 (4.96–5.96)

*AF* atrial fibrillation, *CI* Confidence interval, *CRM* cardio–renal–metabolic, *CV* cardiovascular, *MACE-HF* composite of cardiovascular death, ischemic stroke, myocardial infarction and new-onset heart failure, %*IR* % incidence rate

When stratified by sex, risk of all-cause death and CV death was higher among non-obese, regardless of sex. Conversely, for the MACE-HF and new AF, patient’s sex was of greater importance than the obesity status, with males having higher risk of these outcomes. Kaplan–Meier survival curves detailing these findings are available in Supplementary Figure S2.

### Multivariate analysis

When adjusted for age and sex, both non-obese and obese extremely unhealthy patients had the highest risk of all-cause death with aHRs of 1.12 (95% CI, 1.05–1.19) and 1.12 (95% CI, 1.04–1.20) respectively; CV death with aHRs of 1.75 (95% CI, 1.54–1.99) and 1.89 (95% CI, 1.62–2.19), respectively; and HF with aHRs of 2.13 (95% CI, 2.00–2.27) and 2.66 (95% CI, 2.49–2.84), respectively (Supplementary Figure S3).

Similarly, the risk of stroke and MI (both as a component of MACE-HF), were the highest in the obese extremely unhealthy patients with aHRs of 1.53 (95% CI, 1.29–1.82) and 1.45 (95% CI, 1.24–1.68), respectively (Supplementary Figure S4). In the obese and non- obese extremely unhealthy patients, aHRs were 1.94 (95% CI 1.68–2.25) and 2.15 (95% CI 1.90–2.44), respectively. The risk of MACE-HF, and new-onset AF were the highest in extremely unhealthy obese patients with aHRs of 2.42 (95% CI, 2.28–2.58) and 1.98 (95% CI, 1.80–2.18), respectively.

**Fig. 3:**
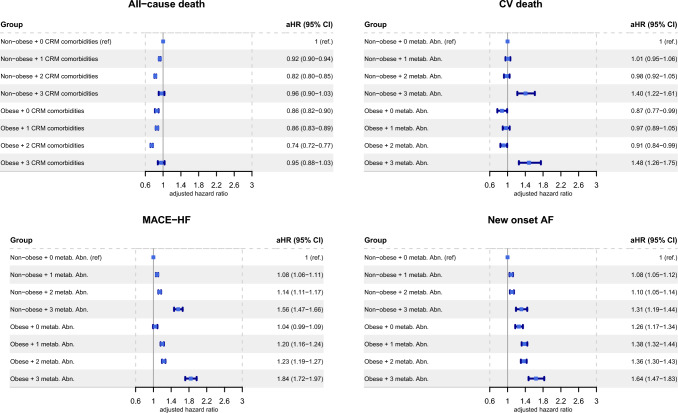
Adjusted hazard ratios for the associations between body size phenotypes and cardio-renal-metabolic comorbidities for all cause-death CV death, MACE-HF, and new-onset AF. Adjustment was made for age, sex, type of diabetes, smoking status, alcohol-related diagnoses, valve disease, coronary artery disease, previous PCI, previous CABG, vascular disease, previous pacemaker or ICD, lung disease, sleep apnoea syndrome, liver disease, thyroid disease, inflammatory disease, anaemia, previous cancer, cognitive impairment and illicit drug uses. *AF* atrial fibrillation, *aHR* adjusted hazard ratio, *CABG* coronary artery bypass grafting, *CI* confidence interval, *CRM* cardio-renal-metabolic, *CV* cardiovascular, *ICD* Implantable cardioverter defibrillator, *MACE-HF* composite of CV death, ischemic stroke, myocardial infarction and new-onset heart failure, *PCI* Percutaneous coronary intervention, *ref*. reference

After further adjustment for multiple covariates (see description of Fig. 3), both non-obese and obese extremely unhealthy patients still had the highest risk of CV death with aHRs of 1.40 (95% CI, 1.22–1.61) and 1.48 (95% CI, 1.26–1.75), respectively, and MACE-HF with aHRs of 1.56 (95% CI, 1.47–1.66) and 1.84 (95% CI, 1.72–1.97), respectively. Obese extremely unhealthy patients had the highest risk of incident AF with aHRs of 1.64 (95% CI, 1.47–1.83).

## Discussion

In the present study using data that were representative for the contemporary French national population, our principal findings are as follows: (i) approx. 70% of DM patients were non-obese; (ii) the highest incidence rate of all cause and CV- death as well as IS and MI was for non-obese, extremely unhealthy group (presenting with 3 cardio-renal-metabolic comorbidities); (iii) the highest incidence of MACE-HF, HF and AF was for both non-obese and obese, extremely unhealthy patients; (iv) the highest adjusted risk of CV- death and MACE-HF were for both non-obese and obese extremely unhealthy patients; and (v) the highest adjusted risk of new AF were for extremely unhealthy obese patients.

Epidemiological data related to obesity prevalence in DM are changing and the numbers are lowering nowadays when compared the data from other countries reported 17 years ago (approx. 50–60%) [17, 18]. For example, in a recent population study from France published in 2023, 788 (41.1%) of patients with known DM were obese [19]. In the present cohort of French patients with DM, the prevalence of obesity was only approx. 30%.

Our data show that non-obese patients with DM were commonly burdened with other cardio–renal–metabolic comorbidities (i.e. hypertension, CKD, hyperlipidemia), consistent with prior French National general population data (i.e. not focused solely on patients with DM) [16]. In everyday clinical scenario, DM itself is often related to other chronic comorbidities with the increasing prevalence of multimorbidity, defined as having two or more long-term conditions [20], especially cardiac, renal and metabolic diseases [21]. DM as well as obesity confers higher CV risk but neither diabetes nor obesity per se are “yes/no” diagnoses in term of CV health where not every patient who has diabetes or obesity presents the same CV risk [22].

There is ongoing controversy whether the obesity paradox exists in DM (9–13) or not [14, 15]. We found that the highest incidence of all-cause and CV- death as well as IS and MI was observed among patients without obesity, but this was linked to being burdened with 3 concomitant cardio–renal–metabolic abnormalities (which we termed an ‘extremely unhealthy’ phenotype). Those who were obese but had no additional comorbidities besides DM had the lowest incidence rate of these outcomes. While these findings might initially appear to support the obesity paradox, this does not reflect long-term health outcomes. Indeed, prior research indicates that obese individuals may have a lower overall life expectancy compared to their leaner counterparts [23, 24]. In addition, short-term follow-up may favor younger, obese patients with fewer immediate comorbidities, yielding more favorable prognosis. Subsequent adjustments for various comorbidities negated any significant difference in all-cause death and CV death risk between obese and non-obese individuals within the ‘extremely unhealthy’ category, with the obese having higher risk of MACE-HF.

Another explanation for the obesity paradox could be that lower body weight in the presence of cardio-renal-metabolic disorders which are to some extent obesity-related may reflect underlying illness that contributes to adverse cardiovascular events. Moreover one of the chronic conditions which was twice more prevalent in the extremely unhealthy phenotype of patients with DM was CKD, which is a well-recognized predictor of increased CV risk per se [25, 26]. When adjusted for age and sex, the extremely unhealthy DM patients, both with and without obesity, presented with higher risks of all cause death, CV- death, HF, IS and MI. The importance of associated comorbidities has been highlighted by Lassale et al. where metabolically unhealthy patients were associated with higher coronary heart disease risk than healthy people, irrespective of their BMI [27].

Obesity however still seems to be an important risk factor for MACE-HF and incident AF because as shown in the current analysis, an obese, extremely unhealthy phenotype was independently associated with the highest risk of these outcomes. Previously obese patients with DM had already been assessed as a group with the highest risk of AF incidence [28, 29]. Non-obese patients were not found to be at increased risk of AF compared to the obese ones, but contrary to our study no comorbidity has been taken into account [29].What is more, obese patients, comparing to non-obese ones, were more burdened with obstructive sleep apnoea syndrome which is one of acknowledged risk factors of poor CV prognosis [30, 31]. The present results draw attention to a group of patients with DM, both obese and non-obese but with additional cardio-renal-metabolic co-morbidities, and an extremely unhealthy phenotype resulting in an increased CV risk.

In our cohort, sex played a differential role in all studied outcomes. While obesity status influenced all-cause and cardiovascular death across both sexes with non-obese individuals being at higher risk of all-cause and CV death, it was less impactful than sex in relation to MACE-HF and new-onset AF. This finding confirms previously observed attenuation of gender differences in cardiovascular mortality [32] but does not explain the higher CV risk among non-obese individuals which definitely needs further investigation. This layered interaction between obesity and sex is further complicated when considering other data. A Norwegian longitudinal study reported that even though men had higher rates of incident AF similarly to our study, the influence of BMI was comparable between sexes with the lowest risk of AF among those with normal weight [33, 34]. Meanwhile, the ACCORD trial indicated a sex-BMI interaction among patients with diabetes, with higher BMI conferring a greater risk of incident AF for men [35].These multifaceted observations collectively accentuate the complex interplay between BMI, diabetes status, and sex in influencing long-term cardiovascular outcomes.

Our study confirms the importance of managing cardio–renal–metabolic status of patients with DM regardless of obesity and proves that cardio–renal–metabolic status modifies the relationship between patients’ body mass related phenotype and risk of CV events. Given that many patients with DM have associated comorbidities and lifestyle factors that should be addressed, this has also led to the evolution of more holistic or integrated multidisciplinary management approaches to improve patient care [36].

### Limitations

This study had several limitations that should be addressed. It was observational in nature and based on administrative data with potential biases related to the study methodology. We based the outcomes on diagnoses obtained from ICD-10 codes so we cannot exclude inaccuracies in the diagnostic codes. On the other hand, disease coding is related to reimbursement that is why it is regularly controlled and expected to be of good quality. The large scale of the presented study may however partly compensate for some of the potential biases. Our statistical adjustments were made for numerous variables, however important for patients with DM confounders, namely recent weight change, abdominal obesity, duration and severity of diabetes could not be assessed due to administrative nature of the database. In addition, the absence of laboratory data in our study precludes the stratification of patients by CKD stage, a variable that could offer additional insights into the relationship between renal function and the outcomes observed. Due to the study design based on the ICD-10 codes we had no information related to overweight patients and so we compared only those who were obese to those who were not obese, although a proportion of the latter could be overweight. Moreover, obesity was defined based on BMI which does not fully capture body composition, especially abdominal distribution of body fat. Also, age-related loss of lean muscle mass and bone (sarcopenia) could be the reason for lower body weight especially in older adults.

## Conclusion

Obese and non-obese patients with DM and three concomitant cardio-renal-metabolic co-morbidities are an ‘extremely unhealthy’ phenotype with the highest adjusted risk of CV death as well as MACE-HF. Focus should be not only be on diabetes or obesity as a single disease but attention at managing any concomitant comorbidities associated with them.

## Supplementary Information

Below is the link to the electronic supplementary material.

Supplementary file1 (DOCX 9131 KB)

## Data Availability

Access to the PMSI (the French hospitalization database) is controlled by the CNIL, the independent national ethics committee protecting human rights in France. Due to the sensitive nature of the database, data sharing is not authorized according to the French legislation.
