# Supplementary material for: Metabolically ‘extremely unhealthy’ obese and non-obese patients with diabetes and the risk of cardiovascular events: a French nationwide cohort study
Source: Clin Res Cardiol. 2023 Dec 4;113(11):1534–43. doi: 10.1007/s00392-023-02344-8 (PMC11493818; doi:10.1007/s00392-023-02344-8)
Supplement: Supplementary file 1 — Supplementary file1 (DOCX 9131 KB) [file 392_2023_2344_MOESM1_ESM.docx]

Supplementary material

Supplementary Table S1. List of ICD-10 codes used to derive diagnoses.

| **Label** | **ICD-10 code** |
| --- | --- |
| Diabetes type 1 | E10 |
| Diabetes type 2 | E11 |
| Other types for diabetes | E13, E14 |
| Hypertension | I10, I11, I12, I13, I14, I15 |
| Chronic kidney disease | N18.3, N18.4, N18.5, T86.1, Z49, Z99.2 |
| Hyperlimidemia | E78 |

|  | |  | | **Non-Obese patients  n=235593 (69.1)** | | | | **Obese patients  n=105456 (30.9)** | | | | |  | |  |
| --- | --- | --- | --- | --- | --- | --- | --- | --- | --- | --- | --- | --- | --- | --- | --- |
|  |  | |  | | | | | |  | | | | |  | |
|  | **Overall** | | **0 CRM comorbidities** | | **1 CRM comorbidities** | **2 CRM comorbidities** | **3 CRM comorbidities** | | **0 CRM comorbidities** | **1 CRM comorbidities** | **2 CRM comorbidities** | **3 CRM comorbidities** | | **P-Value** | |
| **n** | **n=341049** | | **n=67469 (19.8)** | | **n=96223 (28.2)** | **n=63654 (18.7)** | **n=8247 (2.4)** | | **n=13764 (4.0)** | **n=38675 (11.3)** | **n=45532 (13.4)** | **n=7485 (2.2)** | |  | |
| **Age (year)** | 69.1±13.4 | | 64.4±16.7 | | 72.9±12.0 | 72.3±11.1 | 72.3±11.7 | | 59.9±14.9 | 67.1±12.2 | 67.5±10.7 | 70.0±10.2 | | <0.001 | |
| **Sex (male)** | 194784 (57.1) | | 39522 (58.6) | | 55439 (57.6) | 40258 (63.2) | 5455 (66.1) | | 6242 (45.4) | 18612 (48.1) | 25011 (54.9) | 4245 (56.7) | | <0.001 | |
| **Type 1 diabetes Type 2 diabetes Other types of diabetes** | 34783 (10.2) | | 14008 (20.8) | | 8990 (9.3) | 5042 (7.9) | 646 (7.8) | | 1379 (10.0) | 2296 (5.9) | 2132 (4.7) | 290 (3.9) | | <0.001 | |
|  | 302206 (88.6) | | 52118 (77.2) | | 86072 (89.5) | 57929 (91.0) | 7508 (91.0) | | 12198 (88.6) | 36056 (93.2) | 43163 (94.8) | 7162 (95.7) | | <0.001 | |
|  | 4060 (1.2) | | 1343 (2.0) | | 1161 (1.2) | 683 (1.1) | 93 (1.1) | | 187 (1.4) | 323 (0.8) | 237 (0.5) | 33 (0.4) | | <0.001 | |
| **Hypertension** | 239077 (70.1) | | 0 (0.0) | | 81879 (85.1) | 63293 (99.4) | 8247 (100.0) | | 0 (0.0) | 32821 (84.9) | 45352 (99.6) | 7485 (100.0) | | <0.001 | |
| **Hyperlipidemia** | 125918 (36.9) | | 0 (0.0) | | 11775 (12.2) | 52303 (82.2) | 8247 (100.0) | | 0 (0.0) | 5271 (13.6) | 40837 (89.7) | 7485 (100.0) | | <0.001 | |
| **Chronic kidney disease** | 35471 (10.4) | | 0 (0.0) | | 2569 (2.7) | 11712 (18.4) | 8247 (100.0) | | 0 (0.0) | 583 (1.5) | 4875 (10.7) | 7485 (100.0) | | <0.001 | |
| **Smoker** | 34202 (10.0) | | 4983 (7.4) | | 7226 (7.5) | 7689 (12.1) | 1174 (14.2) | | 1312 (9.5) | 3742 (9.7) | 6875 (15.1) | 1201 (16.0) | | <0.001 | |
| **Alcohol-related diagnoses** | 27885 (8.2) | | 6206 (9.2) | | 7495 (7.8) | 4663 (7.3) | 633 (7.7) | | 1073 (7.8) | 3193 (8.3) | 3957 (8.7) | 665 (8.9) | | <0.001 | |
| **Valve disease** | 27913 (8.2) | | 2592 (3.8) | | 7214 (7.5) | 7118 (11.2) | 1492 (18.1) | | 509 (3.7) | 2792 (7.2) | 4806 (10.6) | 1390 (18.6) | | <0.001 | |
| **Coronary artery disease** | 92757 (27.2) | | 8254 (12.2) | | 21999 (22.9) | 25055 (39.4) | 4483 (54.4) | | 1597 (11.6) | 8706 (22.5) | 18380 (40.4) | 4283 (57.2) | | <0.001 | |
| **Previous PCI** | 22676 (6.6) | | 1887 (2.8) | | 4699 (4.9) | 6880 (10.8) | 1138 (13.8) | | 317 (2.3) | 1788 (4.6) | 4888 (10.7) | 1079 (14.4) | | <0.001 | |
| **Previous CABG** | 4506 (1.3) | | 194 (0.3) | | 680 (0.7) | 1458 (2.3) | 276 (3.3) | | 36 (0.3) | 324 (0.8) | 1249 (2.7) | 289 (3.9) | | <0.001 | |
| **Vascular disease** | 75207 (22.1) | | 6102 (9.0) | | 17508 (18.2) | 21419 (33.6) | 4279 (51.9) | | 1131 (8.2) | 6302 (16.3) | 14656 (32.2) | 3810 (50.9) | | <0.001 | |
| **Previous pacemaker or ICD** | 22386 (6.6) | | 2307 (3.4) | | 6400 (6.7) | 5665 (8.9) | 1130 (13.7) | | 355 (2.6) | 2040 (5.3) | 3485 (7.7) | 1004 (13.4) | | <0.001 | |
| **Lung disease** | 59478 (17.4) | | 7314 (10.8) | | 14406 (15.0) | 10430 (16.4) | 1667 (20.2) | | 2519 (18.3) | 9524 (24.6) | 11295 (24.8) | 2323 (31.0) | | <0.001 | |
| **Sleep apnoea syndrome** | 37353 (11.0) | | 1922 (2.8) | | 4648 (4.8) | 3781 (5.9) | 585 (7.1) | | 2364 (17.2) | 9204 (23.8) | 12496 (27.4) | 2353 (31.4) | | <0.001 | |
| **Liver disease** | 26809 (7.9) | | 4819 (7.1) | | 6277 (6.5) | 3885 (6.1) | 552 (6.7) | | 1343 (9.8) | 3953 (10.2) | 5294 (11.6) | 686 (9.2) | | <0.001 | |
| **Thyroid diseases** | 34586 (10.1) | | 4170 (6.2) | | 8363 (8.7) | 6591 (10.4) | 1059 (12.8) | | 1375 (10.0) | 4837 (12.5) | 6860 (15.1) | 1331 (17.8) | | <0.001 | |
| **Inflammatory disease** | 23000 (6.7) | | 3231 (4.8) | | 5965 (6.2) | 4494 (7.1) | 1030 (12.5) | | 702 (5.1) | 2686 (6.9) | 3837 (8.4) | 1055 (14.1) | | <0.001 | |
| **Anaemia** | 53710 (15.7) | | 6643 (9.8) | | 13617 (14.2) | 12327 (19.4) | 3701 (44.9) | | 1226 (8.9) | 5274 (13.6) | 7721 (17.0) | 3201 (42.8) | | <0.001 | |
| **Previous cancer** | 56130 (16.5) | | 10818 (16.0) | | 18030 (18.7) | 11006 (17.3) | 1306 (15.8) | | 1676 (12.2) | 5959 (15.4) | 6302 (13.8) | 1033 (13.8) | | <0.001 | |
| **Cognitive impairment** | 16751 (4.9) | | 2653 (3.9) | | 6531 (6.8) | 3745 (5.9) | 564 (6.8) | | 221 (1.6) | 1316 (3.4) | 1357 (3.0) | 364 (4.9) | | <0.001 | |
| **Illicit drug use** | 1129 (0.3) | | 377 (0.6) | | 243 (0.3) | 148 (0.2) | 38 (0.5) | | 57 (0.4) | 123 (0.3) | 120 (0.3) | 23 (0.3) | | <0.001 | |

Supplementary Table S2. Baseline characteristics of diabetes mellitus patients according to body size phenotypes and number of CRM comorbidities.

Values are n (%) or mean±SD.

CRM: cardio-renal metabolic; PCI: Percutaneous coronary intervention; coronary artery bypass grafting; ICD: Implantable cardioverter defibrillator

|  |  | **Non obese** | | **Obese** | |  |
| --- | --- | --- | --- | --- | --- | --- |
|  | **Overall n=196112** | **Female n=58635 (29.9)** | **Male n=79715 (40.6)** | **Female n=31047 (15.8)** | **Male n=26715 (13.6)** | **P-Value** |
|  |  |  |  |  |  | **P-Value** |
| **Age (year)** | 65.7±13.7 | 67.0±15.6 | 66.7±13.0 | 62.5±13.1 | 63.5±11.0 | <0.001 |
| **Type 1 diabetes Type 2 diabetes Other types of diabetes** | 25092 (12.8) | 9877 (16.8) | 11249 (14.1) | 2266 (7.3) | 1700 (6.4) | <0.001 |
|  | 168406 (85.9) | 47822 (81.6) | 67260 (84.4) | 28528 (91.9) | 24796 (92.8) |  |
|  | 2614 (1.3) | 936 (1.6) | 1206 (1.5) | 253 (0.8) | 219 (0.8) |  |
| **Hypertension** | 121777 (62.1) | 33315 (56.8) | 45490 (57.1) | 22688 (73.1) | 20284 (75.9) | <0.001 |
| **Hyperlipidemia** | 64454 (32.9) | 14466 (24.7) | 22941 (28.8) | 13588 (43.8) | 13459 (50.4) | <0.001 |
| **Chronic kidney disease** | 11721 (6.0) | 3111 (5.3) | 4907 (6.2) | 1836 (5.9) | 1867 (7.0) | <0.001 |
| **Smoker** | 16770 (8.6) | 2707 (4.6) | 8109 (10.2) | 2139 (6.9) | 3815 (14.3) | <0.001 |
| **Alcohol-related diagnoses** | 14527 (7.4) | 2095 (3.6) | 8364 (10.5) | 1039 (3.3) | 3029 (11.3) | <0.001 |
| **Valve disease** | 5275 (2.7) | 1526 (2.6) | 2121 (2.7) | 855 (2.8) | 773 (2.9) | 0.083 |
| **Coronary artery disease** | 24108 (12.3) | 4225 (7.2) | 12052 (15.1) | 2880 (9.3) | 4951 (18.5) | <0.001 |
| **Previous PCI** | 5231 (2.7) | 754 (1.3) | 2947 (3.7) | 422 (1.4) | 1108 (4.1) | <0.001 |
| **Previous CABG** | 561 (0.3) | 49 (0.1) | 304 (0.4) | 36 (0.1) | 172 (0.6) | <0.001 |
| **Vascular disease** | 24697 (12.6) | 4469 (7.6) | 12555 (15.7) | 2762 (8.9) | 4911 (18.4) | <0.001 |
| **Previous pacemaker or ICD** | 3743 (1.9) | 880 (1.5) | 1996 (2.5) | 321 (1.0) | 546 (2.0) | <0.001 |
| **Lung disease** | 23113 (11.8) | 4736 (8.1) | 8795 (11.0) | 5003 (16.1) | 4579 (17.1) | <0.001 |
| **Sleep apnoea syndrome** | 18166 (9.3) | 1597 (2.7) | 3903 (4.9) | 5825 (18.8) | 6841 (25.6) | <0.001 |
| **Liver disease** | 15124 (7.7) | 2773 (4.7) | 5966 (7.5) | 3046 (9.8) | 3339 (12.5) | <0.001 |
| **Thyroid diseases** | 17375 (8.9) | 7521 (12.8) | 2580 (3.2) | 5966 (19.2) | 1308 (4.9) | <0.001 |
| **Inflammatory disease** | 10720 (5.5) | 3382 (5.8) | 3764 (4.7) | 2106 (6.8) | 1468 (5.5) | <0.001 |
| **Anaemia** | 20523 (10.5) | 6536 (11.1) | 7933 (10.0) | 3714 (12.0) | 2340 (8.8) | <0.001 |
| **Previous cancer** | 31200 (15.9) | 7800 (13.3) | 15775 (19.8) | 3264 (10.5) | 4361 (16.3) | <0.001 |
| **Cognitive impairment** | 5760 (2.9) | 2550 (4.3) | 2286 (2.9) | 572 (1.8) | 352 (1.3) | <0.001 |
| **Illicit drug use** | 650 (0.3) | 100 (0.2) | 380 (0.5) | 91 (0.3) | 79 (0.3) | <0.001 |

Supplementary Table S3. Baseline characteristics of diabetes mellitus patients according to body size phenotypes and sex.

Values are n (%) or mean±SD.

CRM: cardio-renal metabolic; PCI: Percutaneous coronary intervention; coronary artery bypass grafting; ICD: Implantable cardioverter defibrillator

|  |  | **Non-Obese patients** | | | | **Obese patients** | | | |  |
| --- | --- | --- | --- | --- | --- | --- | --- | --- | --- | --- |
|  | **Overall** | **0 CRM comorbidities** | **1 CRM comorbidities** | **2 CRM comorbidities** | **3 CRM comorbidities.** | **0 CRM comorbidities** | **1 CRM comorbidities** | **2 CRM comorbidities** | **3 CRM comorbidities** | **P-Value** |
| **All cause death, n (%)** | 61787 (31.5) | 14606 (29.3) | 21034 (38.1) | 10727 (34.7) | 1085 (45.0) | 2052 (19.6) | 6105 (26.8) | 5443 (24.1) | 735 (37.9) | <0.001 |
| **Cardiovascular death, n (%)** | 10981 (5.6) | 2243 (4.5) | 3721 (6.7) | 2167 (7.0) | 266 (11.0) | 303 (2.9) | 1023 (4.5) | 1070 (4.7) | 188 (9.7) | <0.001 |
| **MACE-HF events, n (%)** | 65089 (33.2) | 12468 (25.0) | 19528 (35.4) | 12617 (40.9) | 1317 (54.6) | 2272 (21.7) | 7337 (32.1) | 8464 (37.6) | 1086 (56.0) | <0.001 |
| **Incident AF, n (%)** | 29166 (14.9) | 5364 (10.8) | 9022 (16.3) | 5391 (17.5) | 532 (22.0) | 1086 (10.4) | 3590 (15.7) | 3729 (16.5) | 452 (23.3) | <0.001 |

Supplementary Table S4. Absolute number of all cause death, cardiovascular death, MACE-HF and incident AF according to body size phenotypes and number of CRM comorbidities.

**Supplementary Figure S1.** Kaplan-Meier curves of all cause-death (**A**), CV-death (**B**), MACE-HF (**C**), and new onset AF (**D**) in the whole cohort (n=341,049), without exclusion of those with previous MACE-HF or AF. Log rank p-values all<0.0001. AF: atrial fibrillation; CRM: cardio-renal metabolic; CV: cardiovascular; MACE-HF: composite of cardiovascular death, ischemic stroke; myocardial infarction and new onset heart failure

**Supplementary Figure S2.** Kaplan-Meier curves of all cause-death (**A**), CV-death (**B**), MACE-HF (**C**), and new onset AF (**D**) regarding sex. Log rank p-values all<0.0001. AF: atrial fibrillation; CV: cardiovascular; MACE-HF: composite of cardiovascular death, ischemic stroke; myocardial infarction and new onset heart failure.


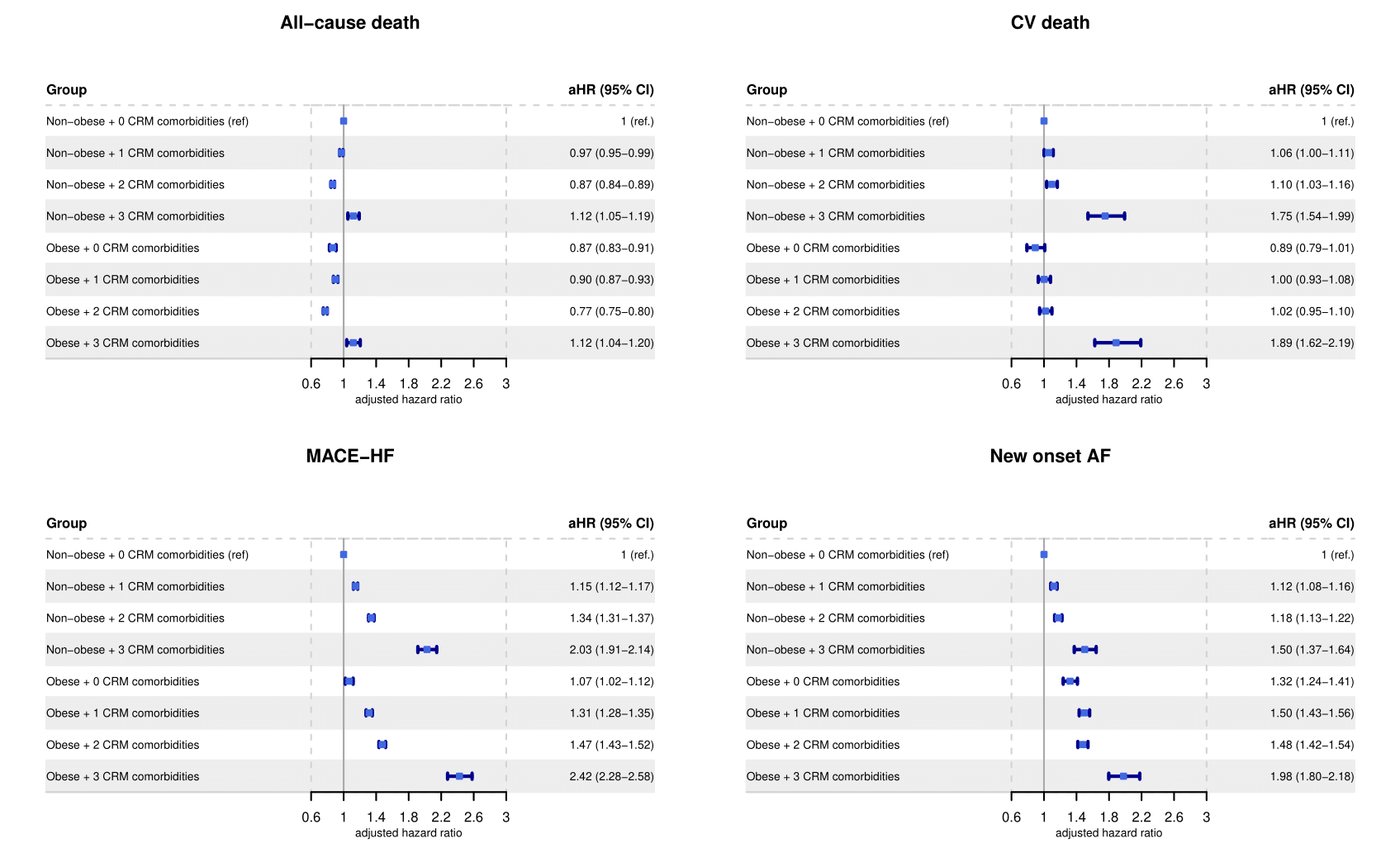


Supplementary Figure S3 Adjusted hazard ratios for the associations between body size phenotypes and CRM status for all cause-death, CV-death, MACE-HF, and new onset AF. Adjustment has been made for age and sex.

AF: atrial fibrillation; aHR: adjusted hazard ratio; CI: confidence interval; CV: cardiovascular; MACE-HF: composite of CV-death, ischemic stroke; myocardial infarction and new onset heart failure; ref.: reference


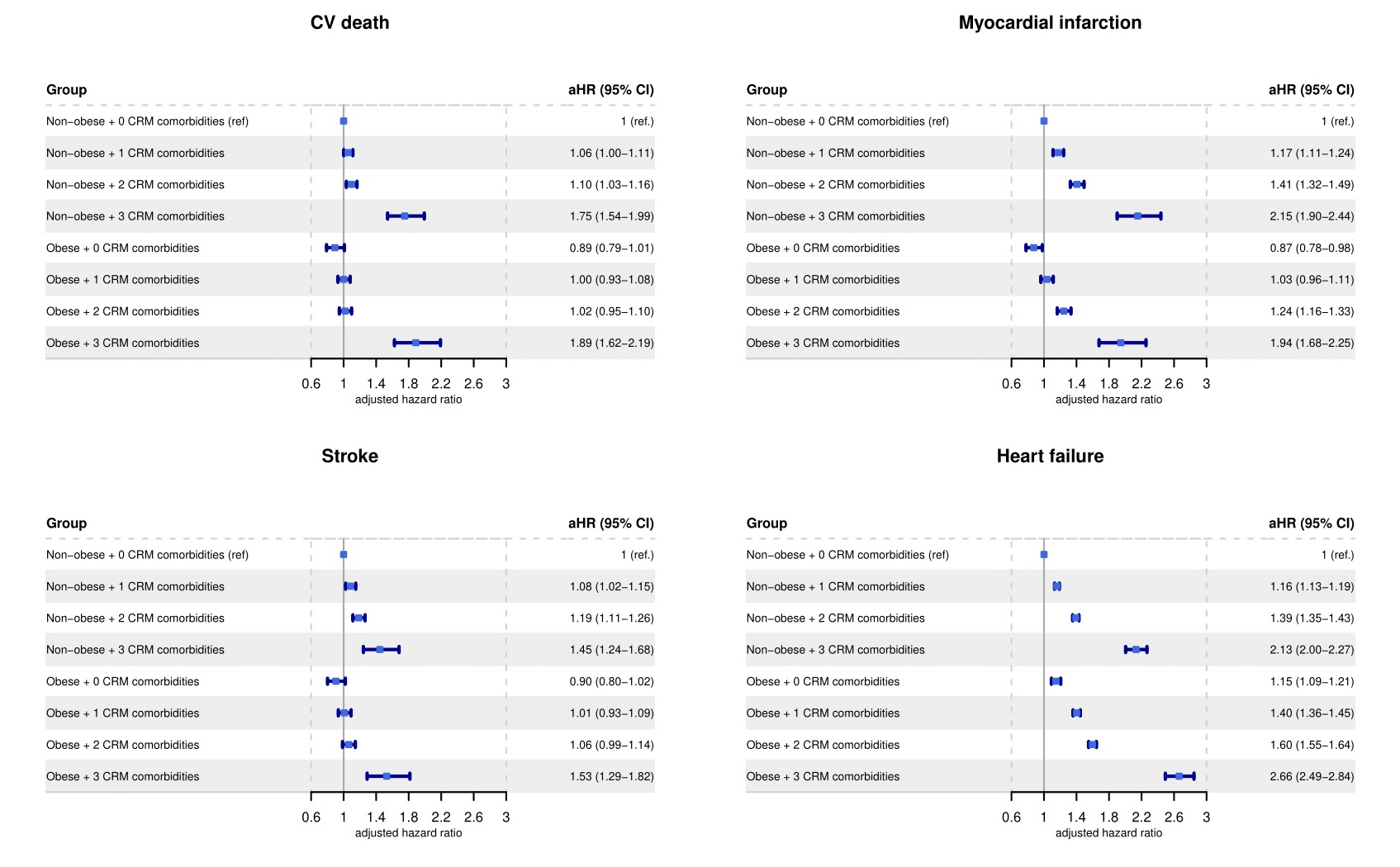


Supplementary Figure S4 Adjusted hazard ratios for the associations between body size phenotypes and CRM status for CV-death, myocardial infarction, stroke and heart failure. Adjustment has been made for age and sex.

AF: atrial fibrillation; aHR: adjusted hazard ratio; CI: confidence interval; CV: cardiovascular; MACE-HF: composite of CV-death, ischemic stroke; myocardial infarction and new onset heart failure; ref.: reference
